# The Anti-Parkinsonism Effects of K_ATP_ Channel Blockade in the 6-Hydroxydopamine-Induced Animal Model: The Role of Oxidative Stress

**DOI:** 10.18869/nirp.bcn.8.3.183

**Published:** 2017

**Authors:** Hossein Piri, Hashem Haghdoost-Yazdi, Negin Fraidouni, Tahereh Dargahi, Mohamadhosein Yaghoubidoust, Abbas Azadmehr

**Affiliations:** 1. Cellular and Molecular Research Center, Qazvin University of Medical Sciences, Qazvin, Iran.; 2. Student Research Committee, School of Medicine, Qazvin University of Medical Sciences, Qazvin, Iran.; 3. Department of Immunology, School of Medicine, Babol University of Medical Sciences, Babol, Iran.

**Keywords:** Parkinson disease, 6-Hydroxydopamine, Glibenclamide, B vitamins, Malondialdehyde

## Abstract

**Introduction:**

Studies suggest that ATP-sensitive potassium (K_ATP_) channels are a potential pharmacotherapeutic target for neuroprotection in neurodegenerative diseases. The current study aimed at evaluating the effect of pretreatment with glibenclamide (Glib) and B vitamins supplement on the severity of behavioral symptoms in 6-hydroxydopamine (OHDA)-induced Parkinsonism. Also malondialdehyde (MDA) concentration was measured in the blood and brain suspensions to find probable neuroprotective mechanism of Glib.

**Methods:**

The 6-OHDA was injected into striatum of rats by stereotaxic surgery. Treatment with Glib and B vitamins was started before the surgery and continued up to 3 weeks after that. Development and severity of Parkinsonism were evaluated by conventional behavioral tests. MDA values were measured spectrophotometrically using thiobarbituric acid and MDA standard curve.

**Results:**

Pretreatments with Glib, at both doses of 1 and 5 mg/kg or B vitamins significantly ameliorated severity of the behavioral symptoms. Pretreatment with a combination of Glib and B vitamins was more effective than pretreatment with Glib or B vitamins alone. Also, pretreatment with B vitamins, Glib, or a combination of them reduced MDA concentration in the brain suspensions. Decrease in MDA concentration in the group of rats that received a combination of B vitamins and Glib was more prominent than that of the Glib groups.

**Conclusion:**

As severity of the behavioral symptoms in the 6-OHDA-induced Parkinsonism reflects the degree of the lesion in Substantia Nigra (SN) dopaminergic neurons, it is suggested that Glib pretreatment has neuroprotective effect against 6-OHDA-induced neurotoxicity. The current study data also showed that this effect may be mediated by antioxidant effect of Glib.

## 1. Introduction

Parkinson Disease (PD) is a common neurodegenerative movement disorder characterized by selective degeneration of dopaminergic (DA) midbrain neurons, mainly in the substantia nigra pars compacta (SNpc). Although the molecular pathogenesis of PD and the basis for selective DA neuronal loss are controversial, studies suggest that mitochondrial dysfunction and oxidative stress play important roles (Jenner and Olanow 1996; Tatton 2000; Du Lau et al., 2005; Hancock et al., 2007).

ATP-sensitive potassium (K_ATP_) channels are suggested as a potential pharmacotherapeutic target for neuroprotection in some neurodegenerative diseases, including PD (Liss and Roeper 2001; Wang, Hu, Yang, Ding, & Hu, 2005). K_ATP_ channels are present in both plasma membrane and mitochondrial inner membrane, and are activated by a decrease in ATP/ADP ratio. They stabilize the membrane potential and mitochondrial matrix volume during ATP decline (Liss and Roeper 2001; Kou, Klorig, & Bloomquist, 2006). K_ATP_ channels are widely distributed in the brain, especially in the cortex, basal ganglia, hippocampus, and hypothalamus (Mourre, Widmann, & Lazdunski, 1990; Dunn-Meynell and Rawson 1998).

The density of mitoK_ATP_ channels in brain neurons is 7-fold higher than that of the heart cells, indicating that these channels play an essential role in the CNS physiology and pathology (Chai, Niu, Sun, Ding, & Hu, 2006). In the brain, level of K_ATP_ channels is higher in the basal ganglia (Mourre et al., 1990; Dunn-Meynell and Rawson, 1998) and studies show that DA neurons in the SNpc have a high density of K_ATP_ channels (McGroarty and Greenfield, 1996). Dopamine, in turn, regulates K_ATP_ channels in the cells dissociated from SN (Greif, Lin, Liu, & Freedman 1995; McGroarty and Greenfield 1996). Also, tolbutamide, a selective K_ATP_ channel blocker, antagonizes D2 receptor agonist-induced hyperpolarization (Roeper, Hainsworth, & Ashcroft, 1990).

Glibenclamide (Glib), a second generation sulfonylurea, exhibits an inhibitory effect on surface and mitochondrial K_ATP_ channels (Busija et al., 2004). Glib suppresses neutrophil migration and chemotaxis following acute inflammatory response by blocking the K_ATP_ channels (Da Silva-Santos, Santos-Silva, Cunha, & Assreuy, 2002; Pompermayer et al., 2007). Glib also has an antioxidant effect, which is independent of its K_ATP_ blocking activity (Nazaroglu, Sepici-Dincel, & Altan, 2009; Al-Azzam, Abdul-Razzak, & Jaradat, 2010). Studies show that Glib ameliorates damage caused by renal and intestinal Ischemic Reperfusion (IR) injury (Pompermayer et al., 2005; Pompermayer et al., 2007). Several authors reported that Glib also provides neuroprotective effects (Lee, Kim, Ko, & Han, 2005; Simard et al., 2006; Toulorge, Guerreiro, Hirsch, & Michel, 2010; Rodriguez-Pallares, Parga, Joglar, Guerra, & Labandeira-Garcia, 2011; Kim et al., 2014).

Regarding PD, however, most of the studies examined either the effect of K_ATP_ channels in cellular models of PD or the role of these channels in regulating neurotransmitters in PD-related brain regions. Few studies that evaluated the effect of K_ATP_ channel blockers on the animal models of PD mainly focused on the effect of posttreatment with these blockers and their pretreatment effect is largely unknown. Therefore, the current study evaluated the effect of pretreatment with Glib on the severity of behavioral symptoms in 6-OHDA-induced Parkinsonism. The study also evaluated the effect of pretreatment with a combination of Glib and B vitamins supplement, because it was shown that B vitamins supplementation can reduce the severity of behavioral symptoms in 6-OHDA-induced Parkinsonism (Haghdoost-Yazdi, Fraidouni, Faraji, Jahanihashemi, & Sarookhani, 2012; Haghdoost-Yazdi et al., 2014). To find probable neuroprotective mechanism of Glib, the study measured the concentration of MDA, which is a biomarker of lipid peroxidation and oxidative stress both in serum and in a suspension prepared from midbrain portion of the brain of rats.

## 2. Methods

### 2.1. Animals and experimental groups

Male Wistar rats, provided from Razi Institute (Karaj, Iran) and weighted 250 to 300 g at the onset of the intervention, were housed in large cages (38×59×20 cm) at a temperature-controlled colony room under 12:12 hours light/dark cycle with free access to tap water and food in the form of pellets. All procedures carried out throughout this project were according to the guidelines of animal experiments of Research Council at Qazvin University of Medical Sciences, Qazvin, Iran.

Animals were divided into 6 experimental groups as follows: control (Con, n=9) without any pretreatment; vehicle (Veh, n=10) received ethanol as the solvent of Glib; B vitamins (B vit., n=9) received a complex of B vitamins 5-fold more than that of normal MEM (minimum essential medium); low Glib (n=9) and high Glib (n=9) received low dose (1 mg/kg) and high dose (5 mg/kg) of Glib, respectively; B vit.+Glib (n=9) received both a high dose of Glib and a complex of B vitamins, 5-fold more than that of normal MEM. Additional B vitamins were dissolved in drinking water as described before (Haghdoost-Yazdi et al., 2012). Ethanol and Glib were daily administrated, intraperitoneally (i.p). In addition, the data of another group of rats (n=8), marked as healthy rats, were also used to analyze the data obtained from rotarod test. This extra group consisted of intact rats subjected to no intervention and did not receive 6-OHDA. All of the B vitamins, Glib, 6-OHDA, and apomorphine were purchased from SIGMA-ALDRICH Company (Germany).

### 2.2. Experimental design

**Figure 1.**
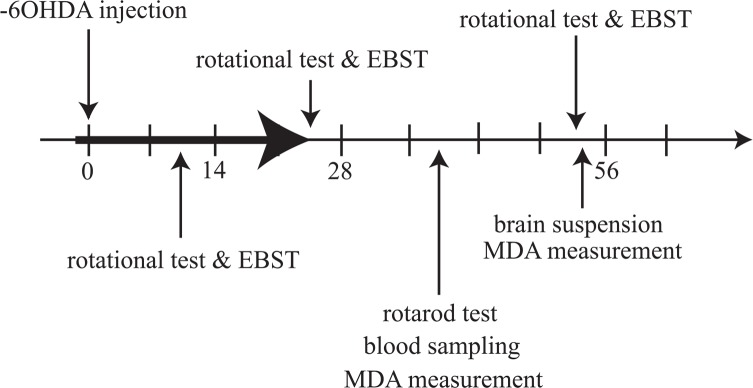
Time schedule used for animal experiments. Animals were tested by apomorphine-induced rotational test and elevated body swing test at 3 times: in the second, fourth, and eighth weeks after 6-OHDA injection. Rotational tests were performed at least 1 hour after termination of the EBST. Rotarod rod test, blood sampling and measurement of its MDA concentration were performed in the 6th week post-surgery. Preparation of the brain suspension and its MDA assay were performed in the eighth week post-surgery. All of pretreatments was started before 6-OHDA injection and continued to 3 weeks after that (black arrow). Numbers show the days after 6-OHDA injection.

All animals (except healthy rats) were subjected to stereotaxic surgery and received 6-OHDA into their brains. All of pretreatments were performed before 6-OHDA injection and continued up to 3 weeks after that (Figure 1). Apomorphine-induced rotational test and elevated body swing test (EBST) were performed during the second, fourth, and eighth weeks post-surgery. Rotarod test was performed during the 6th week post-surgery. Blood sampling and MDA assay were performed after rotarod test. After completion of the behavioral tests, animals were decapitated and MDA concentrations in the brain tissues were measured.

### 2.3. Surgical procedures

Rats were anesthetized by injection of ketamine (100 mg/kg) and xylazine (5 mg/kg), intraperitoneally. Then, 4 μL of 6-OHDA dissolved in isotonic saline containing 0.2 mg/mL of ascorbic acid was administrated into 4 sites in the right striatum using stereotaxic apparatus (Stoelting, USA) by a 10-μL Hamilton syringe. Coordinates for 6-OHDA injections were AP: 1.5, L: −2.5, DV: −6, and AP: 0.8, L: −3, DV: −6, and AP: 0.1, L: −3.2, DV: −6, and AP: −0.5, L: −3.6, DV: −6. AP and L were evaluated from bregma and DV was assessed from the surface of skull according to the atlas of Paxinos and Watson (2007). In the end of surgery, the needle of the Hamilton syringe was left in the brain for an additional 5 minutes and, then, withdrawn at a rate of 1 mm/min.

### 2.4. Behavioral testing

#### 2.4.1. Apomorphine-Induced Rotational Test

Apomorphine-induced rotational test was carried out according to the method described by Fujita et al. (1996). Briefly, animals were 1st habituated for 5 minutes and, then, apomorphine hydrochloride (0.5 mg/kg, i.p., dissolved in saline followed by injection) was administrated. A minute later, the number of rotations was counted for 1 hour in a cylindrical container (28 cm in diameter and 38 cm in height). Contralateral and ipsilateral rotations (far away and toward the lesion side, respectively) were recorded as positive and negative scores and the net number of rotations was calculated by subtraction of the negative scores from the positive ones.

#### 2.4.2. Elevated Body Swing Test

The 6-OHDA treated rats showed swinging behavior biased ipsilateral or contralateral to the lesion side. More biased swings indicated more severe lesion in the Substantia Nigra (SN) dopaminergic neurons. The EBST was carried out according to a method described before (Borlongan, Randall, Cahill, & Sanberg, 1995). Briefly, the animal was habituated for 10 minutes to place all 4 paws on the floor. Then, the animal was held at the base of its tail and lifted up 2 cm above the surface. The neutral position was defined as no deviation of more than 10° to each side. A swing was recorded when the rat moved its body out of the neutral position to each side. Before starting another swing, the rat should be recovered. Swings were counted for a period of 1 minute. During the test, one person held the time and recorded the direction and the number of swings, while another person held the animal. All groups were blind for the procedures. Biased, swings were calculated as follows: L/(L+R) (%) for left-biased swings and R/(R+L) (%) for right-biased swings (L=number of left-biased swings, R=number of right-biased swings).

#### 2.4.3. Rotarod Test

Rotarod test (M.T6800, Borj Sanat, Iran) was performed to test the motor performance and the ability of animal to acquire motor learning. The test was carried out at 6 sessions in 3 consecutive days. Duration of each session was maximum 200 seconds, during which the rotating rod accelerated from 5 to 40 rpm over the first 120 seconds of the session, and remained at maximum speed for the remaining 80 seconds. Animals were scored for their latency (in seconds) to fall (height 30 cm) in each session. To avoid fatigue, 30 minutes rest between trials was given to rats. The data of rotarod test were expressed as the area under the curve (AUC) calculated according to the following formula:

AUC=Time on the rod(s)×[time on the rod(s)×0.44/2]

where 0.44 is the acceleration speed per second.

### 2.5. Blood sampling and preparing brain suspension

Blood samples were collected from the animals’ tail vein using a scalp vein. After clouting, blood samples were centrifuged at 5000 rpm (Eppendorf 5415D) and the sera were stored at −80°C until MDA measurement.

To prepare brain suspension, animals were decapitated under diethyl ether anesthesia, and the brain was removed immediately. Then, the midbrain portion of brain was isolated, washed with normal saline, and sonicated in cooled 1.5% KCl solution to prepare a suspension. Brain suspensions were stored at −80°C until MDA measurement.

### 2.6. MDA measurement

MDA amounts were measured by spectrophotometric method as reported by Albro, Corbett and Schroder (1986). The procedures used thiobarbituric acid (TBA) and MDA standard curve. Reaction of MDA with TBA produced a pink colored solution that had maximum absorbance at 532 nm. The findings were expressed as μM/L for blood samples and μM/g for brain samples.

### 2.7. Statistical analysis

Data were presented as the mean±standard error (SE), in spite of the probable non-normality of the distribution of scores. Data of behavioral tests and MDA were 1st analyzed by Kolmogorov-Smirnov test to determine the normal distribution of data. Since the data did not have normal distribution, they were, then, analyzed by Kruskal–Wallis nonparametric ANOVA followed by a 2-tailed Mann–Whitney U test. A P≤0.05 was considered as statistically significant.

## 3. Results

### 3.1. Rotational behavior

**Figure 2.**
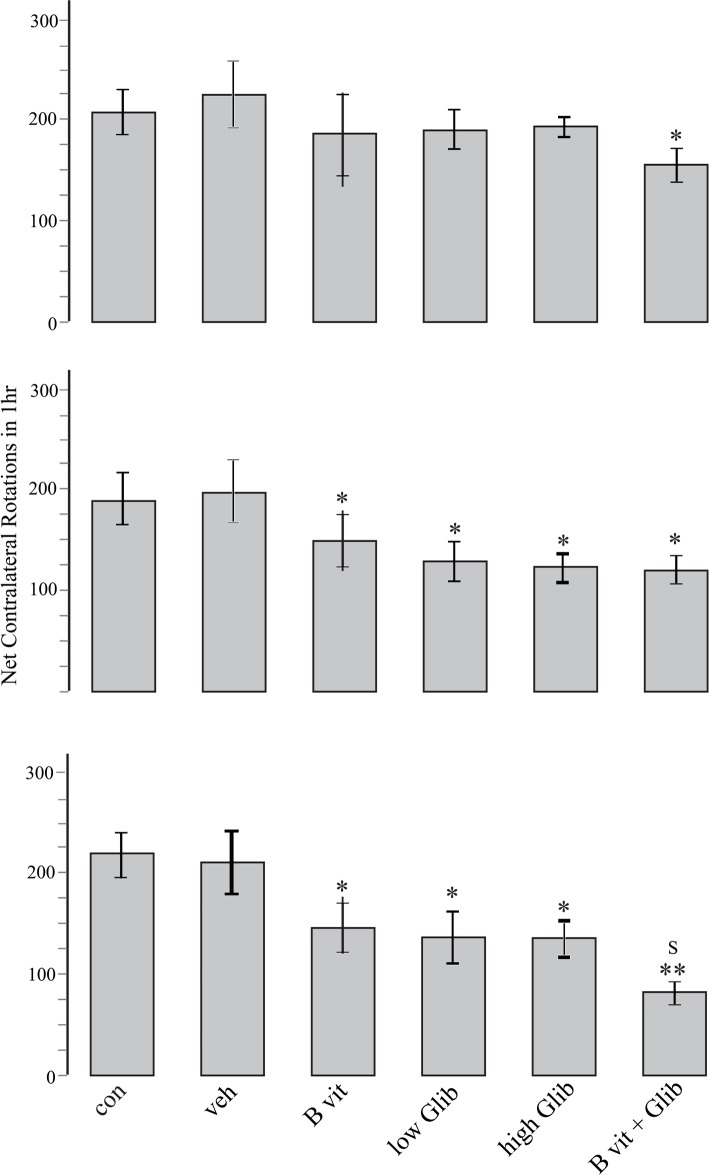
Apomorphine-induced net contralateral rotations of different experimental groups at second (upper plot), fourth (middle plot), and eighth (lower plot) weeks post-surgery. Values are expressed as mean±SE, *P<0.05 and **P<0.01 compared to Veh group; s: P<0.05 compared to B vitamins, low Glib and high Glib groups, Kruskall–Wallis nonparametric test followed by Mann–Whitney U test.

Prominent contralateral (to lesioned side) rotations were observed in all experimental groups indicating that pretreatment with Glib, B vitamins, or a combination of them could not prevent the development of 6-OHDA-induced Parkinsonism. However, as displayed in Figure 2, some pretreatments had significant effects. The most significant effect was observed in B vit.+Glib group. In this group and in all the rotational tests, the number of net contralateral rotations was significantly less than those of the Con and Veh groups. Also, in the third rotational test, the number of rotations in B vit.+Glib group was significantly less than those of B vit. or Glib groups. Pretreatment of rats with both low and high doses of Glib also significantly reduced the number of rotations in the second and third post-surgery tests. Nourishment of rats with B vitamins also ameliorated severity of rotational behavior.

### 3. 2. Swinging behavior

**Figure 3.**
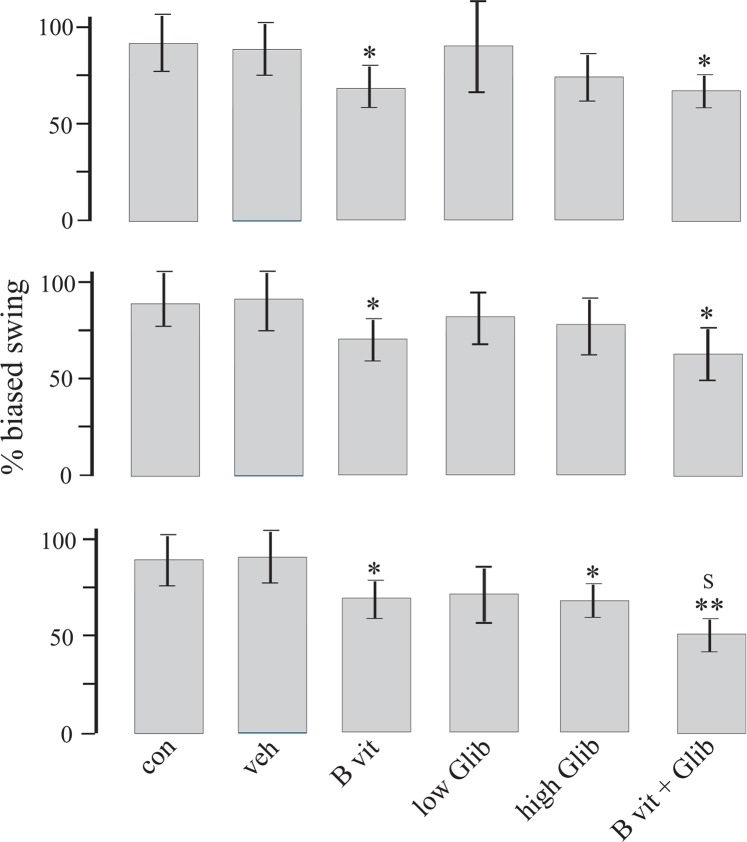
Plots display findings of the EBST in the second (upper plot), fourth (middle plot), and eighth (lower plot) weeks post-surgery. Note 50% means number of left swings was equal to number of right swings. Less than 50% means that most of swings tended toward left (contralateral to lesion side), and more than 50% means that most of swings tended toward right (ipsilateral to lesion side). *P<0.05 and **P<0.01 compared to Veh group; s: P<0.05 compared to low Glib group, Kruskall–Wallis nonparametric test followed by Mann–Whitney U test.

Figure 3 displays findings of EBST. The number of swings varied from 0 to 7 swings and almost all of the 6-OHDA treated rats showed net ipsilateral swings. In B vit.+Glib and B vit. groups, the number of net ipsilateral swings was significantly less than those of the Con and Veh groups. Also, in the third test, the number of net ipsilateral swings in B vit.+Glib group was significantly less than that of the low Glib group. In this test, the number of net ipsilateral swings in high Glib group was significantly less than that of the Veh group.

### 3. 3. Rotarod Test

**Figure 4.**
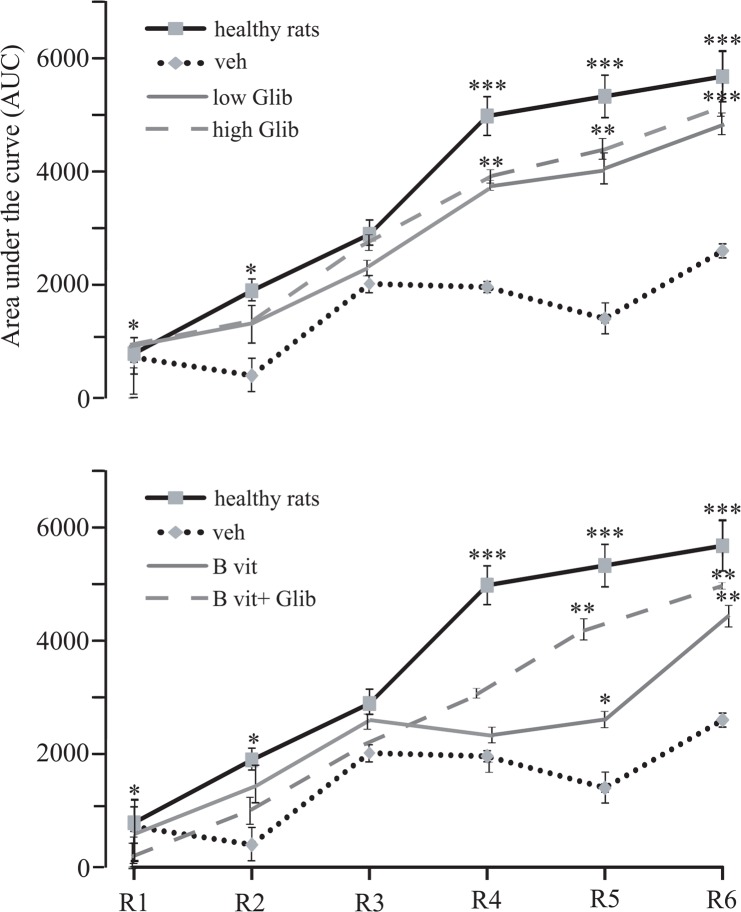
Motor performance of different groups of rats in rotarod test examined at 3 consecutive days, 2 sessions in each. Because Con and Veh groups of rats showed almost similar results, only data of Veh group are shown. Healthy rats rapidly learned how to walk on the rotating rod and reached to maximum performance at fourth session. On the other hand, Parkinsonian rats (Veh group) did not reach to maximum performance and showed weak learning. Values are expressed as mean±SE, *P<0.05; **P<0.01; and ***P<0.001 compared to Veh group; s: P<0.05 compared to B vitamins group, Kruskall–Wallis nonpara-metric test followed by Mann–Whitney U test. AUC: Area under the curve (for more description see the experimental procedures). R1-R6: Sessions of the test; R1: First session; R6: Last session.

Figure 4 quantifies findings of rotarod test of different experimental groups. In the healthy group of rats, the performance improved in consecutive sessions and rats reached the maximum of performance in sessions 4 to 6. All groups of 6-OHDA treated rats also showed some degree of motor learning, but the amount of AUC in them was significantly less than that of the healthy group. In these rats, falling time did not necessarily increase in successive sessions. For example, in Veh group, stepping time on rotarod in the session 5 (R5) was less than those of the 3 (R3) and 4 (R4) sessions. However, significant differences in rotarod performance were observed between the pretreatment and Veh groups. Rats of high Glib, low Glib, and B vit.+Glib groups showed much better learning and AUC in R4, R5, and R6 was significantly higher than that of the Veh group. There was no significant difference between the groups in AUC in the assessed sessions. Also, leaning pattern in these groups was similar to that of the healthy group, and was remarkably better than that of the Veh group. In contrast, learning pattern in B vit. group was more similar to that of the Veh group; in R5, AUC in this group was significantly less than that of the B vit.+Glib group. However, AUC in R5 and R6 in B vit. group were significantly higher than those of Veh group.

### 3. 4. MDA analysis

**Figure 5.**
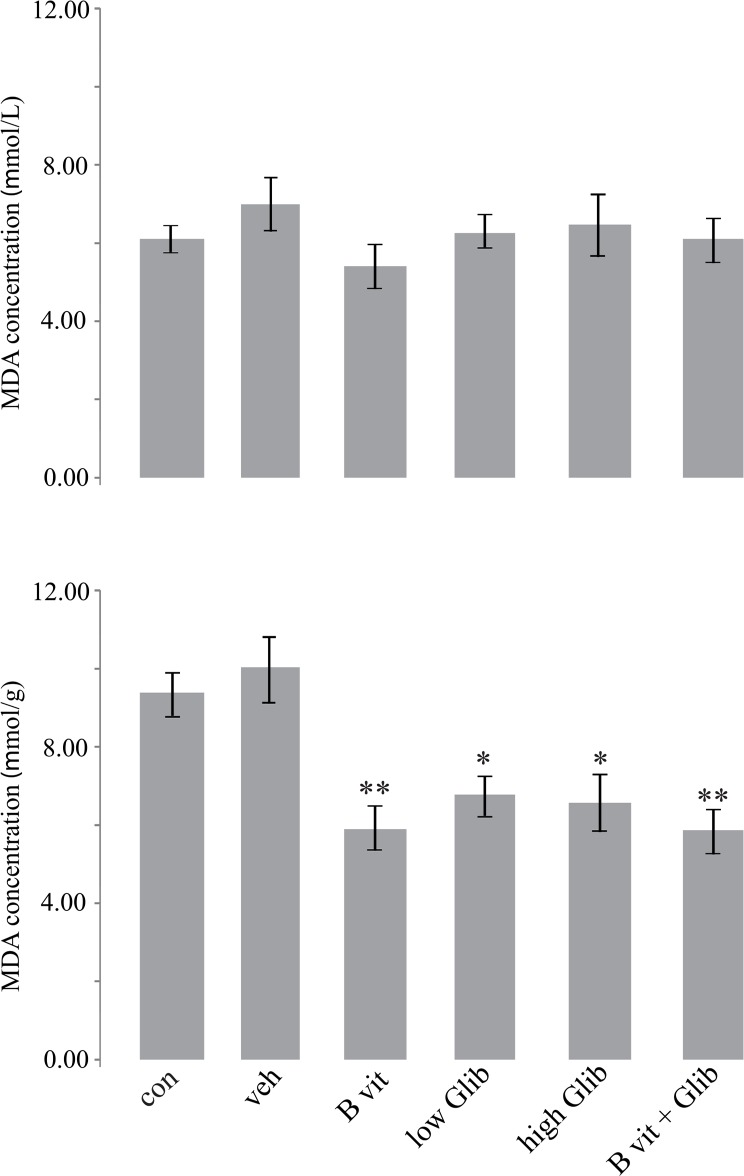
Malondialdehyde concentrations in blood (upper panel) and in a suspension prepared from midbrain portion of the brain (lower panel) of 6-OHDA-treated rats. *P<0.05; **P<0.01 compared to Veh group, Kruskall–Wallis non-parametric test followed by Mann–Whitney U test.

MDA concentration was measured in the blood and in the suspensions prepared from midbrain of rats (Figure 5). In the control group, MDA concentrations in blood and brain suspension were 6.16±0.33 μM/L and 9.34±0.85 μM/g, respectively. In the Veh group, MDA concentrations were higher, but their differences with those of the control group were not significant. Also, there was no significant difference in sera concentrations of MDA between all experimental groups. But, MDA concentration in the brain of all pretreatment groups, especially in B vit. and B vit.+Glib groups was significantly less than that of the Veh group.

## 4. Discussion

The current study provided evidence that pretreatment of rats with Glib ameliorated the severity of behavioral symptoms in 6-OHDA-induced Parkinsonism. Nourishment of rats with B vitamins also ameliorated these symptoms, but pretreatment with a combination of B vitamins and Glib was more effective than either B vitamins or Glib alone. Biochemical data indicated that pretreatment with Glib, B vitamins, or a combination of them reduced MDA concentration in brain tissue, but not in serum. The decrease in MDA in B vit.+Glib and B vit. groups was more obvious than those of the Glib group.

A large body of evidence demonstrated a positive relationship between nigral dopaminergic cell loss and severity of behavioral symptoms in the 6-OHDA treated rats. Rotational test is the most valid test in the evaluation of 6-OHDA-induced dopaminergic cell loss (Borlongan et al., 1995; Dauer and Przedborski 2003; Shimohama, Sawada, Kitamura, & Taniguchi. 2003) and can differentiate the partial lesion from the early complete lesion in SN. Also, falling time in rotarod test inversely correlates with the dopaminergic cell loss in SN (Yuan, Sarre, Ebinger, & Michotte, 2005). In line with this finding, several studies indicated that EBST is a valid behavioral test, which provides an accurate measure of a dopamine-mediated motor function (Borlongan et al., 1995; Abrous et al., 1998; Iancu, Mohapel, Brundin, & Paul, 2005). Based on the available evidence, the current study suggested that pretreatment with Glib and B vitamins provides neuroprotective effect and can reduce the neurotoxic effect of 6-OHDA on the DA neurons in SN.

Similar to the current study results, several studies indicated that inhibition of K_ATP_ channels provided a neuroprotective effect. Lee et al., (2005) showed that Glib attenuated the cytotoxicity of MPP^+^ in PC12 cells by suppressing changes in the mitochondrial membrane permeability. Another study conducted on the rodent models of stroke showed that Glib can reduce cerebral edema infarct volume and mortality by 50% (Simard et al., 2006). Kim et al., (2014) reported that administration of sulfonylureas exerted a neuroprotective effect against kainic acid (KA)-induced hippocampal CA3 neuronal death. Also, studies on the midbrain DA neurons indicated that blockade of K_ATP_ channels protected these neurons against neurodegenerative agents (Toulorge et al., 2010; Rodriguez-Pallares et al., 2012). On the other hand, emerging evidence indicated that opening and activation of K_ATP_ channels, rather than its blockade, protected the neuronal cells against neurotoxins.

Nagy, Kis, Rajapakse, Bari and Busija (2004) reported that K^+^ channel opener diazoxide protected neuronal cells against the toxicity of amyloid β-peptide and glutamate. This effect was inhibited by 5-hydroxydecanoate, a selective mitochondrial K_ATP_ channel inhibitor, and glibenclamide. Several other studies showed that K_ATP_ channel opener of iptakalim provided significant neuroprotection in different animal models of stroke, PD, as well as the cultured cells (Yang et al., 2004; Wang et al., 2005; Yang et al., 2005). This discrepancy in the role of K_ATP_ channels in neuroprotection may arise from the firing nature of neurons. For example, under physiological conditions, DA neurons in midbrain fire spontaneous action potentials and, at least in vitro brain slices, most K_ATP_ channels are closed.

The activation of K_ATP_ channels hyperpolarizes DA neurons, which leads to a complete loss of their normal pacemaker activity. It is suggested that in PD, a chronic reduction of neuronal activity might not be primarily neuroprotective, but might lead to a reduced expression of activity-dependent genes such as neurotrophins that promote survival of neurons (Liss and Roeper 2001). In this scenario, transient K_ATP_ channels activation is a short-term neuroprotective response to metabolic stress, but the activity of chronic K_ATP_ channels could have fatal consequences for the DA neuron. In this regard, Liss et al., (2005) showed that persistent activation of K ATP channels may enhance neurodegeneration. They showed that electrophysiological activity of DA neurons in SN is lost after activation of K_ATP_ channels. Genetic inactivation of the K_ATP_ channels pore-forming subunit Kir6. 2 resulted in rescue of SN dopaminergic neurons in MPTP (1-methyl-4-phenyl-1,2,3,6-tetrahydropyridine) and the mutant weaver mouse models of dopaminergic degeneration. Thus, the activation of K_ATP_ channels can have an unexpected role in promoting death of DA neurons in chronic diseases.

Several mechanisms are suggested for neuroprotective effect of Glib. It is reported that Glib prevents the activation of endothelial caspase-3 through inhibition of the sulfonylurea receptor (SUR)1-regulated NC (Ca-ATP) channels. Caspase-3 activation is described as a major cause of apoptotic processes (Simard et al., 2009). In this regard, Lee et al., (2005) showed that Glib prevents the activation of caspase-3 through suppressing changes in the mitochondrial membrane permeability. Glib also suppresses the inflammatory responses, including the expression of proinflammatory cytokines interleukin (IL)-10 and tumor necrosis factor (TNF)-α (Wali et al., 2012). To confirm this hypothesis, Ortega et al. (2012) reported that Glib fosters microglial neuroprotective activity through the blockade of K_ATP_ channels. Another report suggested that Glib protects cultured midbrain DA neurons by inhibiting a glia-to-neuron signaling cascade that leads to a disruption in calcium homeostasis in the target neurons (Toulorge et al., 2010).

In the current study, to identify possible neuroprotective mechanism of Glib, MDA concentration was measured. MDA is a biomarker of lipid peroxidation and oxidative stress. The current study focused on oxidative stress because 6-OHDA selectively destroys catecholamine neurons by the production of Reactive Oxygen Species (ROS). As pretreatment with Glib reduced MDA concentration in brain tissue, anti-parkinsonian effect of Glib may be mediated by its suppressing effect on the oxidative stress. The current study results were supported by recent reports showing that Glib has antioxidant effects independent from its K_ATP_ blocking activity (Wali et al., 2012). However, the current study data also showed that the effect of B vitamins on the reduction of MDA concentration was more prominent than that of Glib, and was nearly equal to the effect of the combination of Glib and B vitamins. But, the effect of the combination of Glib and B vitamins on the reduction of behavioral symptoms was significantly more potent than those of B vitamins or Glib alone; indicating that Glib might produce anti-parkinsonian effect by other ways too.

In addition to induction of oxidative stress, 6-OHDA induces mitochondrial impairment and ATP deficiency through inhibition of mitochondrial complexes I and IV (Soto-Otero, Méndez-Álvarez, Hermida-Ameijeiras, Muñoz-Patiño, & Labandeira-Garcia, 2002; Blum et al., 2001; Dauer and Przedborski 2003), which can induce neurodegeneration by persistent activation of K_ATP_ channels. Thus, Glib might produce neuroprotective effect by direct blocking of K_ATP_ channels too. Taken together, it is suggested that Glib produced anti-parkinsonian effect by 2 mechanisms: 1) Attenuation of 6-OHDA-induced oxidative stress; and 2) Inhibition of neurodegeneration induced by persistent activation of K_ATP_ channels.

In conclusion, the current study results showed that pretreatment with glibenclamide, a K_ATP_ channel blocker, can ameliorate severity of behavioral symptoms in 6-OHDA-induced Parkinsonism in rats. This effect was augmented by supplementation of rats with B vitamins. As in 6-OHDA treated rats, severity of behavioral symptoms reflected the degree of DA cell loss in SN, it is suggested that Glib pretreatment had neuroprotective effect. Since Glib reduced MDA concentration in brain, it is suggested that at least a part of neuroprotective mechanism of Glib is induced by its antioxidant effect. However, Glib might inhibit 6-OHDA-induced neurodegeneration by prevention of the persistent activation of K_ATP_ channels too.
